# Proteomic analysis of *Escherichia coli* detergent-resistant membranes (DRM)

**DOI:** 10.1371/journal.pone.0223794

**Published:** 2019-10-11

**Authors:** José E. Guzmán-Flores, Lidia Steinemann-Hernández, Luis E. González de la Vara, Marina Gavilanes-Ruiz, Tony Romeo, Adrián F. Alvarez, Dimitris Georgellis

**Affiliations:** 1 Departamento de Genética Molecular, Instituto de Fisiología Celular, Universidad Nacional Autónoma de México, México City, México; 2 Departamento de Biotecnología y Bioquímica, Unidad Irapuato, Cinvestav-IPN, Irapuato, Gto, México; 3 Departamento de Bioquímica, Facultad de Química, Universidad Nacional Autónoma de México, Mexico City, México; 4 Department of Microbiology and Cell Science, IFAS, University of Florida, Gainesville, Florida, United States of America; CINVESTAV-IPN, MEXICO

## Abstract

Membrane microdomains or lipid rafts compartmentalize cellular processes by laterally organizing membrane components. Such sub-membrane structures were mainly described in eukaryotic cells, but, recently, also in bacteria. Here, the protein content of lipid rafts in *Escherichia coli* was explored by mass spectrometry analyses of Detergent Resistant Membranes (DRM). We report that at least three of the four *E*. *coli* flotillin homologous proteins were found to reside in DRM, along with 77 more proteins. Moreover, the proteomic data were validated by subcellular localization, using immunoblot assays and fluorescence microscopy of selected proteins. Our results confirm the existence of lipid raft-like microdomains in the inner membrane of *E*. *coli* and represent the first comprehensive profiling of proteins in these bacterial membrane platforms.

## Introduction

Cell membranes not only confine the boundaries of cells, but also provide highly specialized lipid platforms involved in many cellular processes [1]. For instance, the most studied lipid assemblies of eukaryotic membranes are the lipid rafts, which are liquid-ordered (gel-like) lipid clusters enriched in sphingolipids and cholesterol. Lipid rafts are able to float and diffuse in the lateral plane of the cell membrane and fuse together forming larger aggregates [2]. They provide platforms for the assembly and proper functioning of many protein complexes, which are mainly involved in signal transduction, vesicle trafficking, cytoskeleton rearrangement, and ion channel regulation [3–6]. Cholesterol is known to increase the thickness and to regulate the fluidity of lipid bilayers, and it is considered as an essential lipid component of lipid rafts. Other common constituents of lipid rafts are the flotillins, which belong to a family of proteins that contain the Stomatin/Prohibitin/Flotillin/HflK/C (SPFH) domain. These proteins appear to be essential for the orchestration of processes related to lipid raft formation, and are used as lipid raft markers [4,5,7]. SPFH-domain containing proteins are widely distributed in most bacterial genera. In *Bacillus subtilis* they were found to act as the scaffold for proteins that reside in raft-like membrane microdomains [8]. Moreover, the formation of such membrane microdomains in *B*. *subtillis* was found to be functionally associated with a signaling pathway involved in regulation of biofilm formation and with the Sec protein translocation apparatus [8–11]. However, the membrane *of B*. *subtillis*, like those of most bacteria, does not contain cholesterol, and it has been suggested that other lipids, such as farnesol or farnesol-derived polyisoprenoids, might promote an increased rigidity in the microdomains [12].

Lipid raft-like domains have been also identified in other bacteria, such as *Staphylococcus aureus*, *Borrelia burgdorferi*, *Bacillus anthracis*, *Helicobacter pylori* and *Escherichia coli* [9,13–17]. *B*. *burgdorferi* and *H*. *pylori* possess cholesterol as a membrane component, even though they do not carry out *de novo* sterol biosynthesis. Instead, both bacteria obtain cholesterol from the host epithelial cells to generate glyco-cholesterol derivatives, which are incorporated into the bacterial membranes. Interestingly, both bacterial species appear to form cholesterol-containing membrane microdomains that are assembled into the outer membrane [15,16].

The close packing of lipids in the liquid-ordered phases, typically found in lipid rafts, prevents its solubilization by cold non-ionic detergents. Therefore, the study of lipid rafts, in a variety of eukaryotic and prokaryotic organisms, has been based on the extraction of detergent resistant membranes (DRM). Although detergent resistance in itself does not necessarily reflect preexisting raft domains, results obtained from DRM analysis have often been consistent with those obtained by other approaches, such as direct imaging or functional analysis [18,19]. Thus, DRM isolation provides a useful tool for the study of potential protein-lipid raft associations.

In recent studies, proteomic analyses of DRMs have been carried out in *B*. *subtilis*, *S*. *aureus*, *B*. *burgdorferi* and *H*. *pylori*, and the results suggest that bacterial membrane microdomains, like eukaryotic lipid rafts, play important roles in various cellular processes, such as membrane transport, secretion and virulence [9,16,20–23]. Nevertheless, even though several proteomic analyses of *E*. *coli* membranes have been carried out [24–28], there are no data regarding the composition or protein content of raft-like microdomains from this model bacterium. Here, we report that some 80 proteins, involved in transport, protein secretion, energy metabolism, cell maintenance and signaling, were found to be enriched in DRM. Among these proteins were HflC, HflK, and YbbK (QmcA), three of the four SPFH-containing proteins encoded by the *E*. *coli* genome, that are generally used as lipid raft markers. Thus, the first comprehensive proteomic profile of DRMs from *E*. *coli* is reported, providing information about the cellular processes that may be associated with lipid rafts in this organism.

## Materials and methods

### Bacterial strains, plasmids, and growth conditions

*E*. *coli* strain MG1655 was chosen as the genetic background of all created strains because it is a reference *E*. *coli* strain, and because we recently reported a DRM isolation protocol using this strain [17]. Chromosomal tagging of *hflC*, *hflK*, *qmcA*, *yqiK* and *yidC* genes was achieved by PCR and homologous recombination of the amplification products using the lambda red recombinase system [29,30], resulting in strains IFC5019 (*hflC*::*ha*-Cm^r^) [17], IFC5021 (*hflK*::*ha*-Cm^r^), IFC5022 (*qmcA*::*3xFLAG*-Kn^r^), IFC5023 (*yqiK*::*3xFLAG*-Kn^r^) and IFC5024 (*yidC*::*3xFLAG*-Kn^r^). All oligonucleotides used in PCR amplification reactions are shown in S1 Table. Strain IFC5025 (*hflC*::*ha qmcA*::*3xFLAG yqiK*::*3xFLAG*-Kn^r^) was constructed by two successive transfer steps of the *qmcA*::*3xFLAG*-Kan^r^ and *yqiK*::*3xFLAG*-Kan^r^ alleles from strains IFC5022 and IFC5023 into strain IFC5019 by P1*vir* transduction. In some cases, antibiotic resistance marker was eliminated by expressing the FLP recombinase from plasmid pCP20 [29]. Similarly, strains IFC5026 (*qmcA*::*mCherry*-Kn^r^) and IFC5027 (*yidC*::*mCherry*-Cm^r^) were generated by lambda red recombinase-facilitated homologous recombination of PCR amplified products using primers pair pFluor-ybbK-Fw / pKD-ybbK-Rv and plasmid pMXFL1 [17] as template or yidC-Fluor-Fw / pKD-yidC-Rv and pMXFL2 [17] as template, respectively.

To construct plasmid pMX549, expressing a *glnP*-*mCherry* fusion under the control of the L-arabinose-inducible promoter *ara*, the *glnP* and *mCherry*, coding sequences were PCR amplified using the primer pair Glnp-EcoRI-Fw / Glnp-SacI-Rv and chromosomal DNA from MG1655 as the template, and the primer pair YfpcfSacI / YfPcr1HindIII and plasmid pCHYC-4 [31] as the template, respectively. The two amplified DNA fragments were SacI digested and ligated together, and the product was used as template in a PCR reaction with primers Glnp-EcoRI-Fw and YfPcr1HindIII. Then, purified PCR product was digested with EcoRI and HindIII and cloned into the same restriction sites of pMX020 [32], resulting in plasmid pMX549. To construct plasmid pMX550 (*glnP*-*mCherry*), a 2.7 Kb DNA fragment containing the *ara* promoter and the *glnP*-*mCherry* fusion, obtained from plasmid pMX549 by ClaI and HindIII digestion, was cloned into NruI and HindIII restriction sites of plasmid pACT3 [33]. To construct plasmids pMX551 (*acrA*-*mCherry*), pMX552 (*aas*-*mCherry*) and pMX553 (*rbbA*-*mCherry*), the *acrA*, *aas* and *rbbA* coding DNA sequences were PCR amplified, using the primer pairs Acra-NdeI-Fw / Acra-SacI-Rv, Aas-NdeI-Fw / Aas-SacI-Rv, and Rbba-NdeI-Fw / Rbba-SacI-Rv, respectively, and chromosomal DNA from MG1655 as template, and cloned into NdeI and SacI sites of plasmid pMX550. To construct plasmids pMX554 (*acrA*-*3xFLAG*), pMX555 (*aas*-*3xFLAG*) and pMX556 (*rbbA*-*3xFLAG*), a DNA fragment carrying the 3xFLAG coding sequence upstream the kanamycin resistance cassette was amplified by PCR using primers 3xFLAG-SacI-Fw and 3xFLAG-HindIII-Rv, and plasmid pSUB11 [30] as template. Then, purified PCR product was digested with SacI and HindIII and cloned into the same restriction sites of plasmids pMX551, pMX552 or pMX553, respectively. A schematic work-flow of the above constructed plasmids is presented in S1 Fig. All DNA fragments cloned from PCR-amplified material were sequenced to check that no undesired base changes had been introduced. DNA sequence analysis was performed by the “Unidad de Biologia Molecular” at IFC, UNAM.

*E*. *coli* strains were routinely grown in LB medium at 37°C. When necessary, ampicillin, kanamycin, or chloramphenicol was used at a final concentration of 100, 50 or 25 μg/ml, respectively.

### DRM isolation

DRM fractions were obtained as described previously [17]. Briefly, exponential phase growing *E*. *coli* cells were treated with 10 μg/ml ampicillin to generate filamented cells that were harvested, resuspended in buffer A (1 M sucrose, 0.2 M Tris-HCl [pH 8.0]), and treated with 2 mM EDTA and 12.5 μg/ml lysozyme for 10 min. Next, spheroplasts were obtained by adding sterile water to reach a sucrose concentration of 0.1 M. Spheroplasts, whose formation was confirmed by phase-contrast microscopy, were harvested, resuspended in ice-cold buffer B (20 mM Tris-HCl [pH 7.2], 50 mM NaCl, 5 mM EDTA) containing 20% w/w sucrose, and passed through a French pressure cell. Inner membrane (IM) vesicles were isolated from the spheroplast lysate by ultracentrifugation (~113,000xg) in a discontinuous sucrose gradient (20–50% w/w). IM-containing fractions were pooled and recovered by ultracentrifugation, and 500 μg of protein was mixed with ice-cold Triton X-100 (Pierce, Rockford, IL, USA), resulting in a final detergent concentration of 1% w/v and in a detergent:protein ratio of 8:1, and incubated for 30 min on ice. The DRM fraction were obtained by flotation in a continuous OptiPrep (Axis-Shield, Oslo, Norway) gradient after ultracentrifugation (~173,000xg), concentrated by ultracentrifugation (~106,000xg), and stored at -80°C.

### Protein digestion with trypsin

Proteins in IM or DRM samples were precipitated with trichloroacetic acid (TCA) by adding 100 μl of 10X TE buffer (100 mM Tris-HCl, 10 mM disodium EDTA, pH 8.0) and 100 μl of 72% TCA to samples with 20 μg of total protein. After 2 h of incubation on ice, proteins were pelleted by centrifugation at 14,000 rpm at 4 °C for 10 min, washed with 1 ml of cold acetone and dried at room temperature for 20 min. Precipitated proteins were solubilized in 10 μL of 6 M urea, reduced by with 2.5 μL of reduction buffer (45 mM DTT, 100 mM ammonium bicarbonate) for 30 min at 37 °C, and alkylated by adding 2.5 μL of alkylation buffer (100 mM iodoacetamide, 100 mM ammonium bicarbonate) for 20 min at 24 °C in the dark. Before trypsin digestion, 20 μL of water was added to reduce the urea concentration to 2 M. Protein digestion was performed by adding 10 μL of trypsin solution (5 ng/μL of trypsin sequencing grade from Promega, 50 mM ammonium bicarbonate), samples were incubated at 37 °C for 18 h, and the reaction was stopped by adding 5 μL of 5% formic acid. Protein digests were dried down by vacuum centrifugation and stored at -20 °C.

### LC-MS/MS analysis

Prior to LC-MS/MS analysis, protein digests were resolubilized under agitation in 10 μL of 0.2% formic acid for 15 min. Desalting and cleanup of the samples was done by using C18 ZipTip pipette tips (Millipore, Billerica, MA). Eluates were dried down in a vacuum centrifuge and then resolubilized under agitation in 10 μL of 2% ACN and 1% formic acid for 15 min. The peptide mixture was separated by LC using C18 reversed phase column with a high-pressure packing. A 75 μm i.d. Self-Pack PicoFrit fused silica capillary column (New Objective, Woburn, MA) was packed with the C18 Jupiter 5 μm 300 Å reverse-phase material (Phenomenex, Torrance, CA), and this column was installed on the Easy-nLC II system (Proxeon Biosystems, Odense, Denmark). The separated peptides were directly electrosprayed into a Linear Trap Quadropole (LTQ) Orbitrap Velos (ThermoFisher Scientific, Bremen, Germany) equipped with a Proxeon nanoelectrospray ion source. The solutions used for chromatography were 0.2% formic acid (Solvent A) and 100% ACN/0.2% formic acid (Solvent B). Samples were loaded on-column at a flowrate of 600 nL/min and eluted with a 2-slope gradient at a flowrate of 250 nL/min. Solvent B first increased from 2 to 40% in 100 min and then from 40 to 80% B in 20 min.

LC-MS/MS data acquisition was accomplished using a seventeen-scan event cycle comprised of a full scan MS for scan event 1 acquired in the Orbitrap. The mass resolution for MS was set to 60,000 (at m/z 400) and used to trigger the sixteen additional MS/MS events acquired in parallel in the linear ion trap for the top sixteen most intense ions. Mass over charge ratio range was from 360 to 1700 for MS scanning with a target value of 1,000,000 charges and from ~1/3 of parent m/z ratio to 2000 for MS/MS scanning with a target value of 10,000 charges. The data dependent scan events used a maximum ion fill time of 100 ms and 1 microscan. Target ions already selected for MS/MS were dynamically excluded for 31 s after 2 counts. Nanospray and S-lens voltages were set to 1.3–1.8 kV and 50 V, respectively. Capillary temperature was set to 250 °C. MS/MS conditions were: normalized collision energy, 35 V; activation q, 0.25; activation time, 10 ms.

### Database search

Raw data files of fragmentation spectra were converted to *mzXML* files by RawConverter software tool [34] and compared against the MG1655 *E*. *coli* strain sequence (4,306 entries) of the UniProt database (downloaded on August 18, 2017; Proteome ID: UP000000625), using the Comet search engine in the Trans Proteome Pipeline (TPP) suite [35] with the following parameters: The ion-mass tolerances of monoisotopic peptide precursor and fragmentation products were 3 Da and 1 Da respectively, without correcting for isotope errors. Oxidation of methionines (+16) was considered as the only variable modification, and two missed cleavages were allowed. Peptide assignations were validated with PeptideProphet, filtering out peptides with a probability under 0.7. A protein was considered to be positively identified when ProteinProphet probability was >0.99, and at least three unique peptides were assigned (S2 Table). The spectra count for each inner membrane protein from an IM sample and the average value from two biological replica of DRM fraction were analyzed and used to determine which proteins were enriched in lipid rafts (S3 Table).

### *In silico* analyses

Prediction of number of the transmembrane (TM) domains and signal peptides was carried out using the TOPCONS server (http://topcons.cbr.su.se/) [36]. Lipoprotein signal sequences and lipoprotein inner or outer membrane association were predicted using the LipoP 1.0 Server (http://www.cbs.dtu.dk/services/LipoP/) [37]. Amphipathic in-plane membrane anchor was predicted using Amphipaseek tool of NPS@ server [38].

### Immunoblotting

Equal volumes (10 μl) of representative fractions from OptiPrep gradients were separated by SDS-PAGE (10% polyacrylamide gel), and the proteins were transferred to a Hybond-ECL filter (Amersham Bio-sciences). The filter was equilibrated in TTBS buffer (25 mM Tris, 150 mM NaCl, and 0.1% Tween-20) for 10 min and incubated in blocking buffer (5% w/v milk in TTBS) for 1 h at room temperature. Monoclonal antibodies against HA or 3XFLAG epitope were added at a dilution of 1:10,000 and incubated for 1 h at room temperature. The bound antibody was detected by using anti-mouse IgG antibody conjugated to horseradish peroxidase (Sigma Aldrich) and the Immobilon Western detection system (Millipore). It has to be mentioned that immunodetection of HflC-HA and YbbK-3Flag was carried out on all independently generated membrane fractions, including the ones used for proteomics. Immunodetection of all other protein markers was carried out on at least three independently generated membrane fractions, but not the ones used for proteomic analysis.

### Fluorescence microscopy

*E*. *coli* cells carrying either AcrA-mCherry, YidC-mCherry, HflC-mCherry, QmcA-mCherry, Aas-mCherry or RbbA-mCherry fusion, were grown in LB medium at 37 °C to an optical density at 600 nm (OD_600_) of 1.5, and aliquots of the cell cultures (2 μl) were immobilized on glass slides previously covered with freshly made M9 medium 1% agarose pads [39]. Cells were observed under an upright microscope (Eclipse E600, Nikon) equipped with an oil-immersion objective lens microscope (100x, NA 1.47). mCherry fluorescence was exited with an X-Cite 120 light source system, using a Chroma filter 39010, and images were acquired with a Hamamatsu ORCA-ER cooled-CCD camera controlled with QCapture Pro (version 6.0) software (QImages). Phase contrast and mCherry fluorescence images were taken at 40 ms and 500 ms exposure, respectively, and processed with ImageJ software [40]. Fluorescence images were subjected to background subtraction using a rolling ball radius of 20 pixels, and fluorescence signals were colored in red, before copying the relevant selections to an image editor software.

## Results

### Preparation of DRM fractions from *E*. *coli* membranes

**Fig 1:**
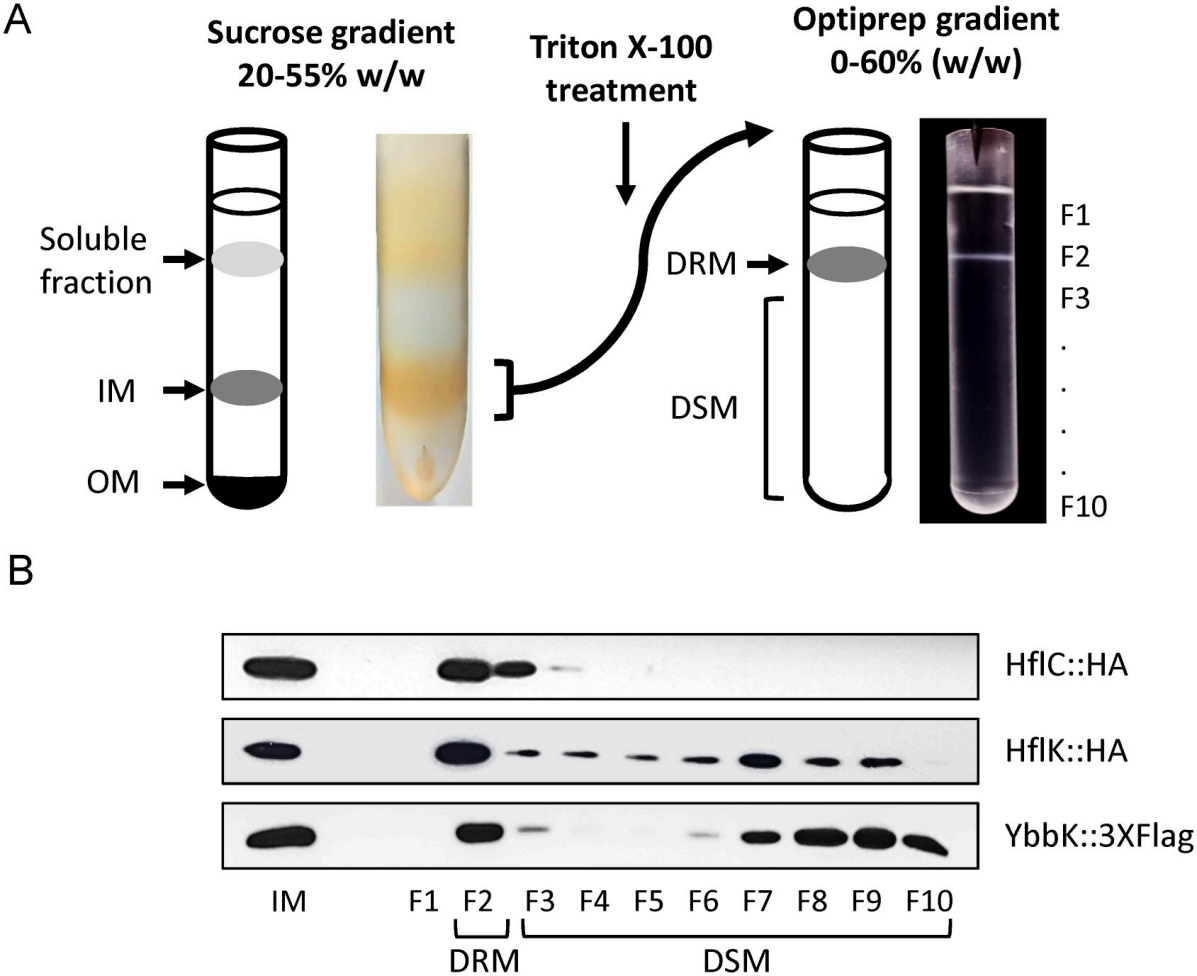
DRM preparation. (A) Schematic illustration of inner-membrane separation by density gradient ultracentrifugation. A spheroplast lysate was loaded on a discontinuous sucrose gradient (20–50% w/w) (cartoon on the left and photography of gradient on the right). The fraction matching to approximately 45% w/w sucrose, corresponding to IM, was separated and treated with ice-cold Triton X-100. The mixture was then loaded on an OptiPrep gradient and ultracentrifuged (cartoon on the left and photography of OptiPrep gradient on the right). Ten 1ml fractions were collected from the top to the bottom of the tube (F1 to F10). DRMs were recovered from F2 whereas DSMs were recovered from lower fractions. (B) IM sample and OptiPrep gradient collected fractions were analyzed by Western blot using specific antibodies against HflC-HA, HflK-HA or QcmA-3xFLAG as described in Materials and Methods section. A representative immunoblot from at least three entirely independent experiments is shown for each protein.

In a recent study, we reported the existence of lipid raft-like microdomains within the plasma membrane of the Gram-negative bacteria *E*. *coli* [17]. The composition and protein cargo of these lipid platforms, however, remain elusive. In order to explore the proteome of these membrane microdomains, we isolated detergent-resistant membranes (DRM), which is the procedure that is typically used for the analysis of lipid rafts of both eukaryotic and prokaryotic cells [19,41]. Because Gram-negative bacteria, such as *E*. *coli*, in addition to the cytoplasmic or inner membrane (IM) are surrounded by an outer membrane (OM), which is naturally resistant to solubilization by detergents [42,43], the use of OM-free IM as the starting material is required if pure DRM are to be obtained [17]. A schematic illustration for DRM isolation is presented in Fig 1A. Briefly, giant spheroplasts of strains IFC5025 and IFC5021, harboring chromosomal HA- or FLAG-tagged hybrids of the four known SPFH-domain proteins of *E*. *coli*, namely HflC, HflK, YqiK and QmcA (YbbK), were generated and lysed by passing them through a French Press. IMs were isolated by ultracentrifugation in discontinuous sucrose gradients, and treated with cold Triton X-100 at a detergent:protein ratio of 8:1. Finally, detergent treated IMs were separated by ultracentrifugation in OptiPrep gradients. DRMs, because of their low density, are able to float on density gradients [44], and, therefore, the visible opaque band settled in the upper part of the gradient (Fig 1A), containing the DRM fraction, was recovered and stored for further analysis. It is worth mentioning that the DRM protein content represented approximately 10% of the initial protein in IM. Proteins in detergent-sensitive membranes (DSM), present in the lower fractions of the gradient were also recovered for immunoblotting assays.

Subsequently, the above collected fractions were probed for their content of the SPFH-domain proteins, namely HflK, HflC, QmcA and YqiK. This was performed because proteins containing the SPFH-domain have been shown to be associated with DRMs in both eukaryotic and bacterial membranes, and are therefore used as lipid raft markers [3,9]. Immunoblot analysis revealed that HflC, HflK and QmcA were partitioned principally into the DRM fraction (Fig 1B), whereas YqiK, which was marginally detected in IM, was not detected along the OptiPrep gradient (not shown). It is likely that the low *yqiK* expression results in not-detectable amounts of YqiK protein in DRM fractions. Interestingly, QmcA, and at a lesser extent HflK, were also detected in DSM fractions, suggesting that the conditions used to obtain DRMs were stringent enough to avoid false positives. Alternatively, populations of membrane rafts with different rigidity may exist, such that these proteins partition into both raft and non-raft membrane regions, depending on the cell physiology, as previously reported [45]. Nevertheless, the presence of the three membrane raft-marker proteins in the DRM fraction corroborates the suitability of our procedures for DRM isolation.

### Proteomic analysis of DRM fraction from *E*. *coli*

**Fig 2:**
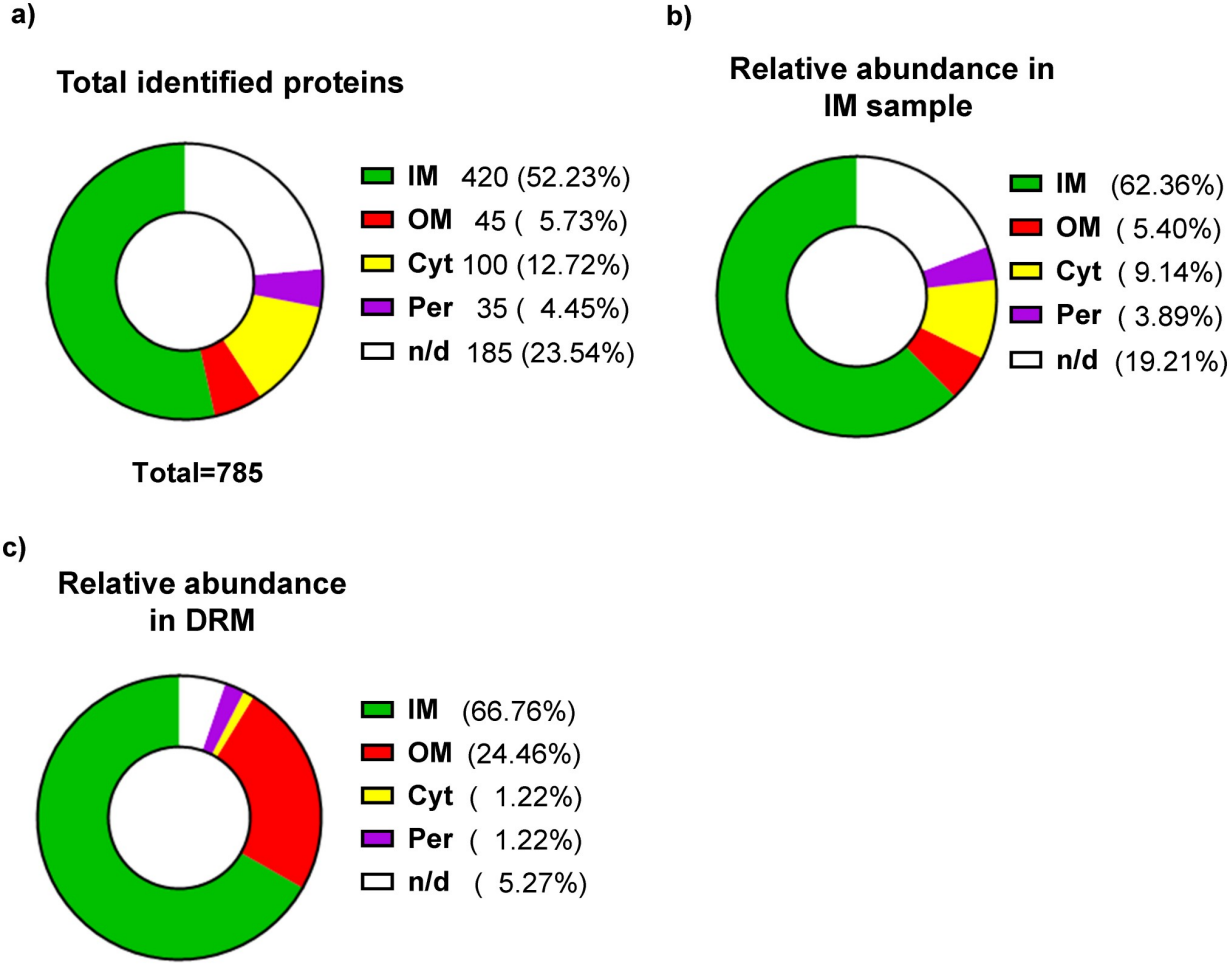
Distribution of identified proteins according to subcellular location. (A) Total proteins identified in inner membrane and DRM samples. (B) Relative abundance of peptides in IM sample. (C) Relative abundance of peptides in DRM samples. IM: Inner membrane proteins, OM: outer membrane proteins, Cyt: cytosolic proteins, Per: periplasmic proteins, n/d: proteins without localization data.

To identify proteins residing in DRM fractions, LC-MS/MS analyses of the obtained IMs and DRMs were conducted. The obtained results were filtered by excluding proteins represented by less than three unique peptides, resulting in the identification of 785 proteins. These proteins were grouped depending on their subcellular localization according to the Uniprot database (S2 Table). Even though the IMs were separated from the OMs and cytosolic proteins, the portion of proteins annotated as IM residents was only 52.23% (420 proteins) of the identified proteins (Fig 2A). On the other hand, 5.73% (45 proteins) corresponded to OM proteins, whereas 12.72% (100 proteins) and 4.45% (35 proteins) represented cytosolic and periplasmic proteins, respectively. The remaining 23.54% (185 proteins) represented proteins with unknown localization (Fig 2A). Inspection of the relative abundance of spectra indicated that the number of peptides corresponding to IM proteins in DRM were slightly higher than in the IM sample (66.76% and 62.36%, respectively), whereas peptides corresponding to OM proteins were greatly enriched (~ 5 times) in DRM in comparison to the IM (24.46% and 5.40%, respectively) (Fig 2B and 2C). This is expected because of the intrinsic detergent-resistance of OMs. In contrast, the amount of peptides corresponding to proteins with cytosolic or periplasmic localization diminished significantly in DRM as compared to the IM (1.22% and 9.14%, respectively for the cytosolic proteins and 1.22 and 3.89%, respectively for the periplasmic proteins) (Fig 2B and 2C). It is probable that some cytosolic or periplasmic proteins could weakly associate with the IM, and were released after detergent treatment. It has to be mentioned that approximately 19% and 5% of the identified peptides in IM and DRM, respectively, corresponded to proteins with unknown localization, and therefore the above mentioned relative abundance of proteins may vary.

**Fig 3:**
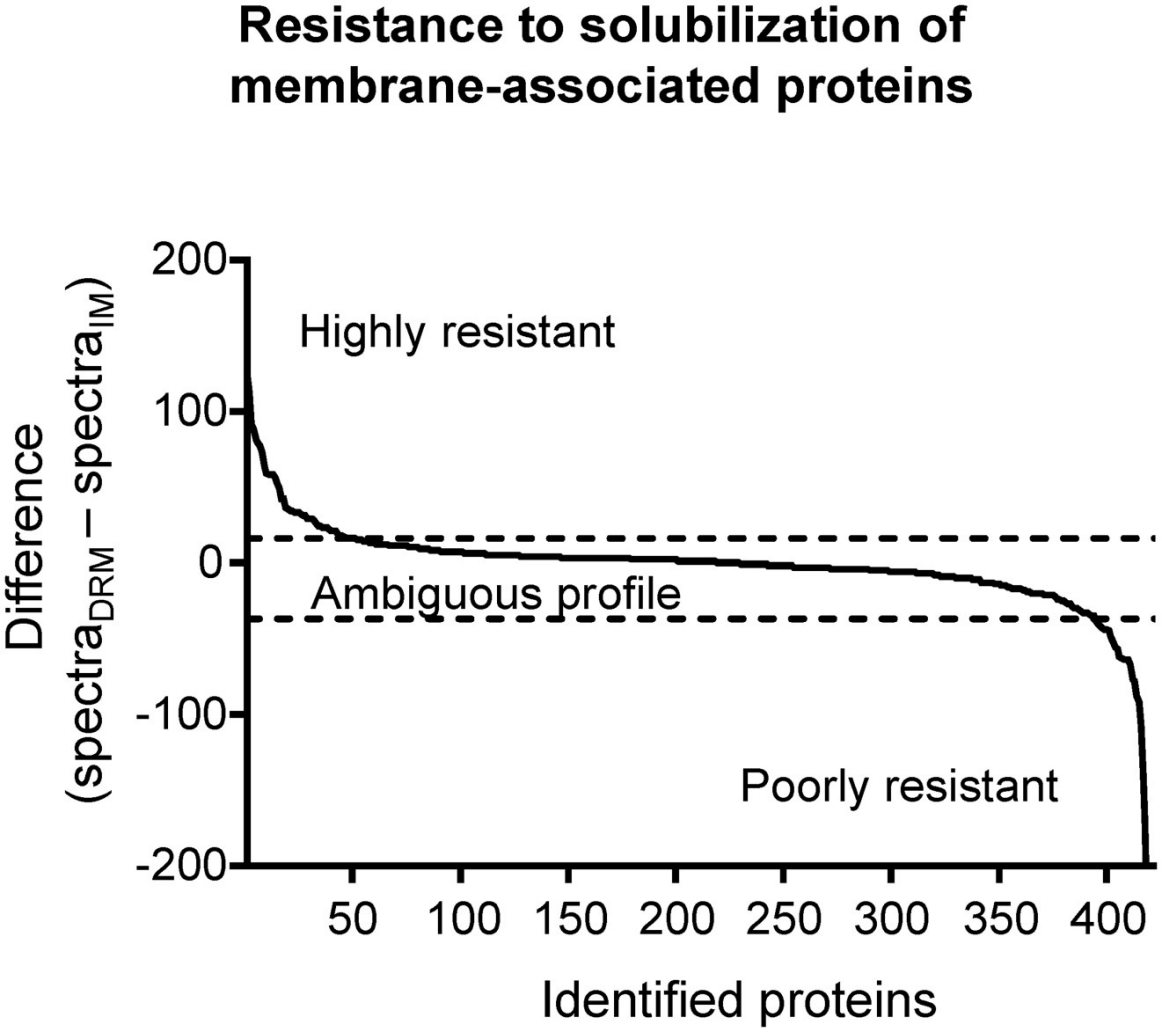
Distribution of identified IM associated proteins according to their detergent solubilization resistance profile. The resistance profile of each protein was determined by calculating the difference between spectra counts obtained from DRM and IM samples. A cut-off of at least 10 positive difference spectra (+10) was used to identify a potential lipid raft associated protein, resulting in 80 selected proteins.

Next, we examined which of the identified proteins were enriched in DRM, suggesting that they may be components of lipid raft-like membrane microdomains. To this end, the spectral counts obtained from the IM sample were subtracted from the ones obtained from the DRM sample, for each of the 420 IM identified proteins. We argued that if, for a given protein, the difference is positive, this protein may reside in a membrane with higher resistance to the detergent treatment, and, therefore, could be considered as a possible resident of membrane microdomains. On the other hand, if the difference is negative, it would suggest that the protein is located in a membrane sensitive to detergent treatment and, therefore, would not be considered as DRM resident. The distribution of the spectra differences for the 420 IM-proteins is shown in Fig 3 and the values in S3 Table. Subsequently, proteins with a difference of less than 10 were excluded and only proteins with a difference of 10 or higher were considered as putative lipid raft components, permitting us to identify 80 proteins (Tables 1–7). As expected, among these proteins were found the SPFH-containing proteins HflK, HflC and QmcA. A functional classification of the identified proteins shows that the *E*. *coli* membrane rafts were mainly enriched in proteins involved in membrane transport, energy metabolism, cell wall metabolism, secretion, and, to a lesser extent, in signaling and scaffolding, reminiscent to those observed for other bacterial membrane microdomains [9,16,20–23]. Noteworthy, among the 80 DRM-enriched proteins, 17 lacked an apparent transmembrane segment. This could be due to their association with membrane-anchored proteins, as is the case for AcrA, which interacts with AcrB on its periplasmic face, forming a multidrug efflux pump complex. Alternatively, periplasmic proteins carrying a lipoprotein signal peptide (SPII) can be covalent linked to membrane lipids by their N-terminal. Our prediction indicates that 8 of the 17 soluble proteins harbor this lipoprotein signal peptide. Finally, proteins could be attached to the membrane by amphipathic helices (in-plane membrane helices, or IPM), which appear to be present in almost half of the total DRM-enriched proteins (Tables 1–7).

**Table 1:** Transporter proteins.

Protein name	Uniprot entry name	Max coverage	IM	DRM	Diff.	Ratio	TM	SP	IPM
Multidrug efflux pump subunit AcrB	ACRB	48.30%	68	191	123	2.81	12	No	Yes
Multidrug efflux pump subunit AcrA	ACRA	81.40%	132	220	88	1.66	0	SPII (IM)	No
PTS system mannitol-specific EIICBA component	PTM3C	67.20%	70	161	91	2.30	7	No	Yes
Multidrug resistance protein MdtF	MDTF	38.10%	25	83	58	3.32	12	No	No
Alpha-ketoglutarate permease	KGTP	30.80%	9	40	31	4.44	12	No	Yes
Paraquat-inducible protein B	PQIB	62.80%	21	52	31	2.45	1	No	Yes
Lipid A export ATP-binding/permease protein MsbA	MSBA	45.00%	38	67	29	1.76	6	No	No
L-cystine transport system ATP-binding protein YecC	YECC	60.40%	4	33	29	8.13	0	No	No
PTS system glucose-specific EIICB component	PTGCB	54.70%	46	75	29	1.62	10	No	Yes
Magnesium transport protein CorA	CORA	74.70%	37	61	24	1.65	2	No	Yes
Nitrate/nitrite transporter NarK	NARK	33.00%	11	35	24	3.14	12	No	No
Multidrug resistance protein MdtE	MDTE	76.40%	41	64	23	1.56	0	SPII (IM)	No
PTS system glucitol/sorbitol-specific EIIB component	PTHB	42.30%	4	23	19	5.63	5	No	Yes
PTS system N-acetylglucosamine-specific EIICBA component	PTW3C	50.20%	58	76	18	1.31	12	No	Yes
Probable glutamate/gamma-aminobutyrate antiporter	GADC	18.20%	11	27	16	2.41	12	No	Yes
D-methionine-binding lipoprotein MetQ	METQ	84.10%	125	140	15	1.12	0	SPII (OM)	No
Glycerol-3-phosphate transporter	GLPT	29.20%	35	49	14	1.39	12	No	No
Lipoprotein 28	NLPA	56.20%	12	25	13	2.08	0	SPII (IM)	No
Probable aminoglycoside efflux pump	ACRD	12.30%	4	16	12	4.00	12	No	No
Macrolide export protein MacA	MACA	41.50%	10	22	12	2.15	1	No	Yes
Putative cation/proton antiporter YbaL	YBAL	24.70%	7	19	12	2.64	13	No	Yes
Serine transporter	SDAC	32.90%	14	25	11	1.79	11	No	No
Proline/betaine transporter (Proline porter II) (PPII)	PROP	17.80%	24	35	11	1.46	12	No	Yes
Carbon starvation protein A	CSTA	15.70%	3	14	11	4.67	16	No	No
Low-affinity inorganic phosphate transporter 1	PITA	27.70%	13	24	11	1.81	10	No	Yes

**Max coverage**, maximum percentage of coverage of identified protein; **IM**, spectra from inner membrane sample; **DRM**, average spectra obtained from DRM samples; **Diff**, average difference between spectra counts in DRM and in IM samples; **Ratio**, average ratio between spectra counts in DRM and in IM samples; **TM**, Predicted transmembrane domains; **SP**, predicted signal peptide [SPI, signal peptide; SPII (IM), lipoprotein signal peptide (predicted inner membrane–specific lipoprotein); SPII (OM), lipoprotein signal peptide (predicted outer membrane–specific lipoprotein); **IPM**, predicted amphipathic in-plane membrane helices.

**Table 2:** Energy biosynthesis related proteins.

Protein name	Uniprot entry name	Max coverage	IM	DRM	Diff.	Ratio	TM	SP	IPM
Cytochrome bo(3) ubiquinol oxidase subunit 2	CYOA	67.60%	36	148	112	4.11	2	No	No
NAD(P) transhydrogenase subunit beta	PNTB	40.00%	25	102	77	4.06	9	No	Yes
NAD(P) transhydrogenase subunit alpha	PNTA	70.00%	145	208	63	1.43	5	No	No
Respiratory nitrate reductase 1 beta chain	NARH	83.60%	120	178	58	1.48	0	No	Yes
Respiratory nitrate reductase 1 alpha chain	NARG	70.70%	313	365	52	1.17	0	No	Yes
Cytochrome bd-I ubiquinol oxidase subunit 1	CYDA	45.00%	108	143	35	1.32	9	No	Yes
Hydrogenase-2 large chain (HYD2)	MBHM	80.60%	107	141	34	1.31	0	No	Yes
Cytochrome bo(3) ubiquinol oxidase subunit 1	CYOB	15.50%	23	45	22	1.93	15	No	Yes
Hydrogenase-2 small chain (HYD2)	MBHT	76.30%	44	65	21	1.48	1	SPI	No
Respiratory nitrate reductase 2 beta chain	NARY	23.00%	6	24	18	4.00	0	No	No
Thiol:disulfide interchange protein DsbD	DSBD	32.00%	8	25	17	3.06	8	SPI	Yes
Electron transport complex subunit RsxG	RSXG	52.40%	4	17	13	4.13	0	SPI	No
Cytochrome c biogenesis ATP-binding export protein CcmA	CCMA	66.70%	3	15	12	4.83	0	No	No
Cytochrome bd-I ubiquinol oxidase subunit 2	CYDB	27.40%	9	21	12	2.28	9	No	Yes
Cytochrome bo(3) ubiquinol oxidase subunit 3	CYOC	20.60%	2	13	11	6.25	5	No	Yes

**Max coverage**, maximum percentage of coverage of identified protein; **IM**, spectra from inner membrane sample; **DRM**, average spectra obtained from DRM samples; **Diff**, average difference between spectra counts in DRM and in IM samples; **Ratio**, average ratio between spectra counts in DRM and in IM samples; **TM**, Predicted transmembrane domains; **SP**, predicted signal peptide [SPI, signal peptide; SPII (IM), lipoprotein signal peptide (predicted inner membrane–specific lipoprotein); SPII (OM), lipoprotein signal peptide (predicted outer membrane–specific lipoprotein); **IPM**, predicted amphipathic in-plane membrane helices.

**Table 3:** Lipid modification and metabolism proteins.

Protein name	Uniprot entry name	Max coverage	IM	DRM	Diff.	Ratio	TM	SP	IPM
Probable phospholipid ABC transporter-binding protein MlaD	MLAD	95.10%	70	119	49	1.69	1	No	No
Phosphoethanolamine transferase EptC	EPTC	45.80%	25	58	33	2.32	5	No	Yes
Uncharacterized lipoprotein YfhM	YFHM	41.70%	35	68	33	1.93	0	SPII (IM)	No

**Max coverage**, maximum percentage of coverage of identified protein; **IM**, spectra from inner membrane sample; **DRM**, average spectra obtained from DRM samples; **Diff**, average difference between spectra counts in DRM and in IM samples; **Ratio**, average ratio between spectra counts in DRM and in IM samples; **TM**, Predicted transmembrane domains; **SP**, predicted signal peptide [SPI, signal peptide; SPII (IM), lipoprotein signal peptide (predicted inner membrane–specific lipoprotein); SPII (OM), lipoprotein signal peptide (predicted outer membrane–specific lipoprotein); **IPM**, predicted amphipathic in-plane membrane helices.

**Table 4:** Response to stimulus proteins.

Protein name	Uniprot entry name	Max coverage	IM	DRM	Diff.	Ratio	TM	SP	IPM
Modulator of FtsH protease HflK	HFLK	75.20%	40	96	56	2.39	1	No	Yes
Modulator of FtsH protease HflC	HFLC	62.90%	80	122	42	1.53	1	No	Yes
Prophage lipoprotein Bor homolog	BORD	58.80%	4	32	28	8.00	0	SPII (OM)	No
Transcriptional activator CadC	CADC	49.60%	2	18	16	9.00	1	No	Yes
Protein QmcA	QMCA	49.50%	8	22	14	2.75	1	No	Yes
Sensor kinase CusS	CUSS	25.20%	5	15	10	2.90	2	No	Yes

**Max coverage**, maximum percentage of coverage of identified protein; **IM**, spectra from inner membrane sample; **DRM**, average spectra obtained from DRM samples; **Diff**, average difference between spectra counts in DRM and in IM samples; **Ratio**, average ratio between spectra counts in DRM and in IM samples; **TM**, Predicted transmembrane domains; **SP**, predicted signal peptide [SPI, signal peptide; SPII (IM), lipoprotein signal peptide (predicted inner membrane–specific lipoprotein); SPII (OM), lipoprotein signal peptide (predicted outer membrane–specific lipoprotein); **IPM**, predicted amphipathic in-plane membrane helices.

**Table 5:** Cell maintaining proteins.

Protein name	Uniprot entry name	Max coverage	IM	DRM	Diff.	Ratio	TM	SP	IPM
Cell division protein DamX	DAMX	81.10%	65	138	73	2.12	1	No	No
Mechanosensitive channel MscK	MSCK	42.60%	50	108	58	2.15	11	SPI	Yes
Peptidoglycan D,D-transpeptidase FtsI	FTSI	59.50%	33	75	42	2.27	1	No	No
Penicillin-binding protein 1B	PBPB	45.10%	32	68	36	2.11	1	No	Yes
Peptidoglycan D,D-transpeptidase MrdA	MRDA	42.80%	17	43	26	2.53	1	No	No
Serine endoprotease DegS	DEGS	62.80%	23	46	23	1.98	0	SPI	No
Cell division protein FtsN	FTSN	73.40%	29	50	21	1.71	1	No	Yes
ECA polysaccharide chain length modulation protein	WZZE	65.80%	17	38	21	2.21	2	No	Yes
3-deoxy-D-manno-octulosonic acid transferase	KDTA	54.80%	17	33	16	1.91	3	No	Yes
Lipopolysaccharide export system permease protein LptG	LPTG	48.10%	5	21	16	4.10	6	No	Yes
Cell division protein FtsQ	FTSQ	61.60%	17	32	15	1.85	1	No	No
Lipopolysaccharide export system protein LptC	LPTC	54.50%	10	23	13	2.30	0	SPI	No
Co-chaperone protein DjlA (DnaJ-like protein DjlA)	DJLA	47.20%	12	23	11	1.92	2	No	Yes
Protein TolQ	TOLQ	44.30%	29	40	11	1.38	3	No	Yes
Protein TolR	TOLR	31.70%	8	19	11	2.31	1	No	No
Cytoskeleton protein RodZ	RODZ	39.20%	22	32	10	1.43	1	No	Yes

**Max coverage**, maximum percentage of coverage of identified protein; **IM**, spectra from inner membrane sample; **DRM**, average spectra obtained from DRM samples; **Diff**, average difference between spectra counts in DRM and in IM samples; **Ratio**, average ratio between spectra counts in DRM and in IM samples; **TM**, Predicted transmembrane domains; **SP**, predicted signal peptide [SPI, signal peptide; SPII (IM), lipoprotein signal peptide (predicted inner membrane–specific lipoprotein); SPII (OM), lipoprotein signal peptide (predicted outer membrane–specific lipoprotein); **IPM**, predicted amphipathic in-plane membrane helices.

**Table 6:** Secretion system proteins.

Protein name	Uniprot entry name	Max coverage	IM	DRM	Diff.	Ratio	TM	SP	IPM
Sec translocon accessory complex subunit YajC	YAJC	87.30%	207	286	79	1.38	1	No	No
Membrane protein insertase YidC	YIDC	58.40%	56	115	59	2.04	5	SPI	Yes
Apolipoprotein N-acyltransferase	LNT	46.70%	9	41	32	4.56	8	No	Yes
Protein-export membrane protein SecG	SECG	55.50%	17	31	14	1.82	2	No	No
Protein translocase subunit SecY	SECY	45.80%	19	31	12	1.61	10	No	No

**Max coverage**, maximum percentage of coverage of identified protein; **IM**, spectra from inner membrane sample; **DRM**, average spectra obtained from DRM samples; **Diff**, average difference between spectra counts in DRM and in IM samples; **Ratio**, average ratio between spectra counts in DRM and in IM samples; **TM**, Predicted transmembrane domains; **SP**, predicted signal peptide [SPI, signal peptide; SPII (IM), lipoprotein signal peptide (predicted inner membrane–specific lipoprotein); SPII (OM), lipoprotein signal peptide (predicted outer membrane–specific lipoprotein); **IPM**, predicted amphipathic in-plane membrane helices.

**Table 7:** Unknown function proteins.

Protein name	Uniprot entry name	Max coverage	IM	DRM	Diff.	Ratio	TM	SP	IPM
Uncharacterized protein YebT	YEBT	69.90%	20	101	81	5.03	1	No	No
Inner membrane protein YejM	YEJM	49.30%	24	58	34	2.40	5	No	Yes
Putative membrane protein IgaA	IGAA	33.80%	9	42	33	4.67	5	No	No
Inner membrane protein YhcB	YHCB	68.90%	35	58	23	1.66	1	No	No
Uncharacterized lipoprotein YbjP	YBJP	65.50%	4	20	16	4.88	0	SPII (OM)	No
Uncharacterized lipoprotein YajG	YAJG	51.00%	4	19	15	4.63	0	SPII (OM)	Yes
Chain length determinant protein	WZZB	77.00%	55	66	11	1.19	2	No	No
Probable inner membrane protein Smp	SMP	46.30%	6	16	10	2.67	1	SPI	No
Low conductance mechanosensitive channel YnaI	YNAI	40.20%	11	21	10	1.91	5	No	Yes
Putative transport protein YbjL	YBJL	29.60%	4	14	10	3.50	11	No	Yes

**Max coverage**, maximum percentage of coverage of identified protein; **IM**, spectra from inner membrane sample; **DRM**, average spectra obtained from DRM samples; **Diff**, average difference between spectra counts in DRM and in IM samples; **Ratio**, average ratio between spectra counts in DRM and in IM samples; **TM**, Predicted transmembrane domains; **SP**, predicted signal peptide [SPI, signal peptide; SPII (IM), lipoprotein signal peptide (predicted inner membrane–specific lipoprotein); SPII (OM), lipoprotein signal peptide (predicted outer membrane–specific lipoprotein); **IPM**, predicted amphipathic in-plane membrane helices.

### DRM-enriched proteins localize in membrane foci in *E*. *coli* cells

**Fig 4:**
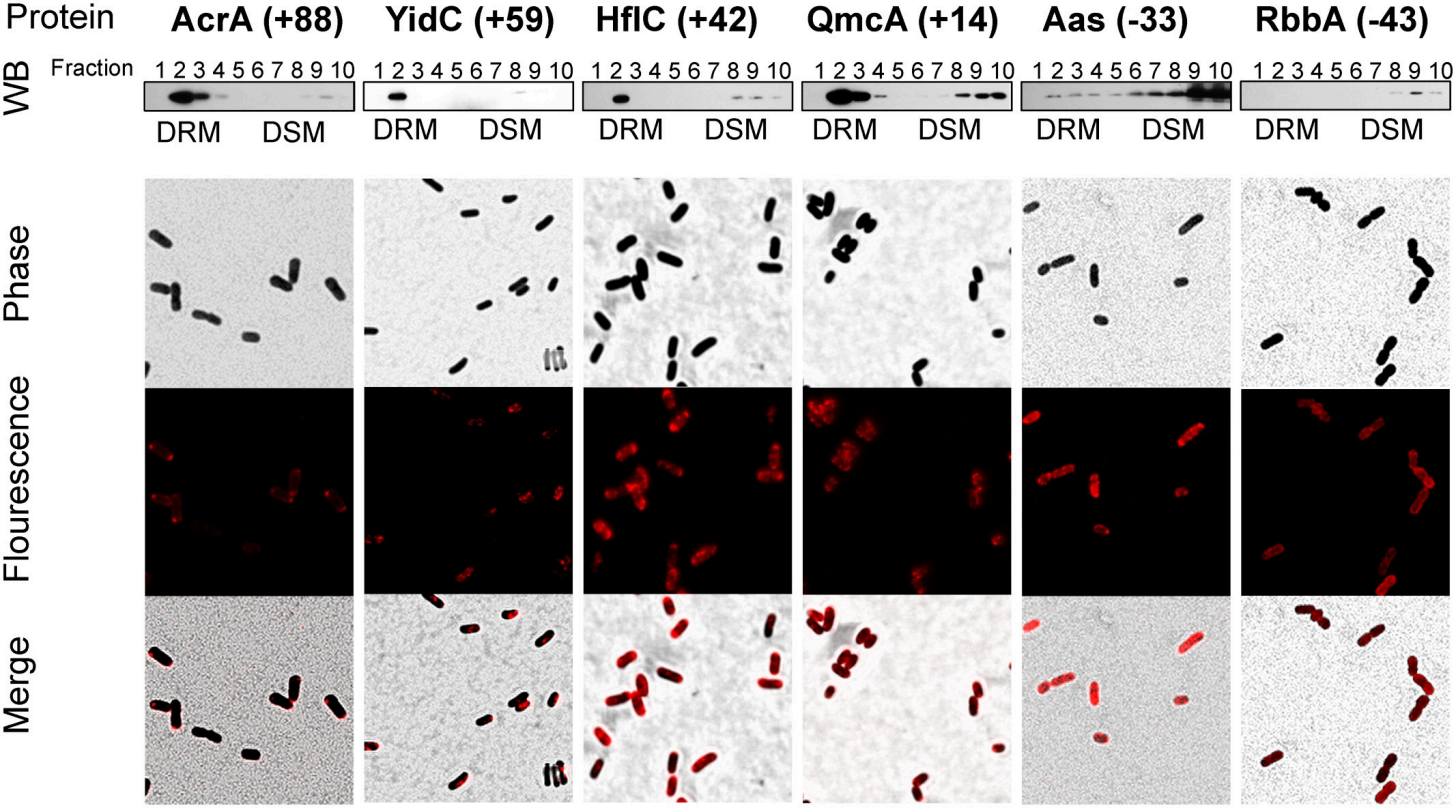
Distribution in OptiPrep gradients and *in vivo* localization of selected lipid raft associated proteins. Top panels: Western blot analysis of DRM (F2) and DSM fractions (F3 to F10), obtained from strains carrying either the AcrA-3xFLAG, YidC-3xFLAG, HflC-HA, QcmA-3xFLAG, Aas-3xFLAG or RbbA-3xFLAG coding sequence allele. A representative immunoblot from at least three entirely independent experiments is shown for each protein. Bottom panels: phase contrast microscopy (top), fluorescence microscopy (middle) and merge (bottom) of living *E*. *coli* cells carrying either AcrA-mCherry, YidC-mCherry, HflC-mCherry, QmcA-mCherry, Aas-mCherry or RbbA-mCherry are shown. Values in parentheses are differences in spectra counts between DRM and IM samples.

To investigate whether the DRM-enriched proteins displayed expected localization patterns within membrane foci, C-terminal mCherry fusions of 6 proteins, 4 of which exhibited a high detergent resistant profile (AcrA, YidC, QmcA and HflC) and 2 not identified in DRM fractions (Aas and RbbA), were generated, and their localization in live cells were detected by epifluorescence microscopy. HflC was used as a control because it contains the SPFH-domain (lipid raft-marker) and its polar localization was previously determined [17]. It was observed that AcrA-mCherry and YidC-mCherry accumulated on the poles of cells, similarly to HflC-mCherry (Fig 4), indicating that these proteins are lipid raft residents. Also, the mCherry fusion of QmcA, another lipid raft-marker protein that was enriched in DRM fractions, appeared as discrete foci with both polar and lateral localization. In contrast, both Aas-mCherry and RbbA-mCherry, which were not identified in DRM fractions, were distributed throughout the membrane, reinforcing the suggestion that these proteins were not associated with lipid raft microdomains. Then, we examined the detergent resistance profile of the membranes in which the above selected proteins are located. To this end, cells carrying 3xFLAG- fusions were obtained and used for IM isolation and Triton X-100 treatment. After separation in OptiPrep gradients, fractions were analyzed by Western blot analysis using anti-Flag antibodies. As expected, AcrA-3xFLAG, YidC-3xFLAG, QmcA-3xFLAG and HflC-3xFLAG were mainly found in the floating DRM fractions, whereas Aas-3xFLAG and RbbA-3xFLAG were present in the soluble fractions at the bottom of gradients. Thus, these results indicate that proteins with higher differences in the number of spectra counts between DRM and IM could be associated to lipid raft-like membrane microdomains in *E*. *coli*, validating our proteomic analysis and their identification as membrane microdomain-residents.

## Discussion

During the past 10 years increasing attention has been drawn to the study of lipid raft-like structures in bacterial membranes. Such membrane microdomains have been identified in *B*. *subtilis*, *S*. *aureus*, *B*. *burgdorferi*, *H*. *pylori* and *E*. *coli* [9,13,15–17]. The herein presented results provide the first comprehensive profile of the lipid raft proteome of *E*. *coli*, providing information on membrane protein organization in this important model bacterium. An important step in our approach was the isolation of *E*. *coli* inner membranes prior to detergent treatment, in order to minimize the contamination of outer membrane proteins. This is because OMs resist detergent treatment and coincide at the same floatation density in OptiPrep gradients. In fact, a ~ 5% of OM-protein contaminants in the isolated IMs were still found in our proteomic analysis, similarly to that reported in previous studies [24,25,28]. As expected, these contaminants were notably enriched in the DRM fractions. For interpreting our mass-spectrometry data, a difference of 10 or higher between spectra obtained from DRM and IM samples (spectraDRM—spectra_IM_ ≥ 10) was used to assign lipid raft residency to each protein. In total, 80 proteins were identified as DRM components, and among them were found three of the four *E*. *coli* SPFH-containing proteins that are typically used as lipid raft markers. Using the above criterion, the location in DRM of proteins producing many spectra (*i*.*e*. most abundant proteins) is emphasized, whereas proteins poorly represented in the proteomic analysis, although important, may be neglected. An alternative approach could be the use of the ratio between peptide counts in DRM and IM samples. For instance, a ratio of 1.5 or higher (spectra_DRM_ / spectra_IM_ ≥1.5) could be used as a criterion to determine enrichment of a protein in DRM. In this case, several proteins (>75) that were discarded in the above analysis could qualify as possible microdomain components, while only 14 of the previously deemed DRM-located proteins failed to meet this criterion. However, proteins with few peptides identified in IM could mislead the interpretation of the proteomic analysis. Taking this into account, 17 additional proteins could be designated as putative lipid raft components if values of spectra_DRM_ / spectra_IM_ ≥1.5 and spectraDRM—spectra_IM_ >5 were considered (S4 Table). Interestingly, YqiK, the only SPFH-containing protein that was not identified by our proteomic analysis, belongs to a group of membrane proteins that has been shown to be particularly elusive to identification by mass spectrometry [25].

In addition to the SPFH-domain containing proteins (lipid raft markers), proteins committed to protein secretion and membrane insertion (e.g. SecY, SecG, YajC, YidC) were identified as putative raft constituents, comparable to those reported for *B*. *subtilis*, *B*. *burgdorferi*, S. *aureus* [20–22]. Likewise, proteins with transport functions were well represented in DRM fractions. These include members of the superfamily of ATP-binding cassette (ABC) transporters, multidrug efflux complexes, and members of the family of phosphoenolpyruvate-dependent sugar phosphotransferase systems (sugar-PTS). This is consistent with previous studies that have identified transporters as common components of lipid microdomains in *B*. *subtilis*, *B*. *burgdorferi* [20,21]. Moreover, the location and function of several eukaryotic ABC transporters, involved in multidrug resistance, have been found to be controlled by their presence in lipid rafts from tumor cells [46]. Interestingly, the ABC transporter MsbA, an essential protein involved in the translocation of lipid A-core from the inner leaflet to the outer leaflet of the IM [47], exhibits a high similarity to mammalian multidrug resistance proteins (MDR) [48]. MDR-1, a member from the latter, which shares a 30% amino acid sequence identity and 46% similarity to MsbA, was also found to be structurally and functionally associated with lipid rafts [49]. Thus, the association of MsbA and other ABC transporters with raft-like microdomains in *E*. *coli* could highlight the co-evolution of transporter complexes and lipid rafts, establishing a suitable microenvironment for proper transport functions.

On the other hand, *E*. *coli* complexes involved in energy metabolism exhibited differential resistance detergent profiles. For instance, all identified components of cytochrome *bo*_3_ and cytochrome *bd* terminal oxidases were enriched in DRM, indicating that these complexes are lipid raft residents. In contrast, NADH-quinone oxidoreductase I components (NuoJ, NuoA, NuoM, NuoH, NuoN, NuoL, NuoB and NuoI), the NADH-quinone oxidoreductase II (Ndh), and components of the ATP-synthase complex, which are functionally associated with terminal oxidases in the electron transport chain, were not enriched in DRM fractions, suggesting that components of a given metabolic pathway could be differentially partitioned into lipid rafts in a point of time.

In spite of the widely established idea that the lipid rafts are implicated in the orchestration of processes related to signal transduction in both eukaryotes and prokaryotes [9,19,50], the only two bacterial histidine kinases associated with lipid rafts, so far, are KinC and WalK from *B*. *subtilis* and *S*. *aureus*, respectively [9]. Here, we identified the sensor kinase of the CusSR two-component system, which regulates the expression of genes involved in copper uptake, as a lipid raft resident. Also, the histidine kinase DcuS, which participates in the control of expression of genes involved in the C4 dicarboxylates catabolism, was found to be enriched 7.5-fold in DRM in comparison with IM. However, this protein was not selected as a raft resident due to its low spectra representation, which was only 1 and 7.5 peptides from IM and DRM, respectively. The absence of bacterial sensor kinases in proteomic analyses of lipid rafts may be due to their very low expression, hampering their identification in membrane samples [25]. Thus, the organization of signaling pathways involving sensor kinases into membrane microdomains remains to be determined.

Although the proteomic analysis of DRMs may provide important data regarding the characteristics of lipid rafts, it is important to emphasize that proteins whose localization in DRM fractions was not validated by experimental approaches must be interpreted with caution. For instance, to support the results of our proteomic analysis, we probed the distribution of selected proteins in live cells, by fluorescence microscopy. As expected, proteins that were enriched in DRM were observed as discrete foci, principally in cell poles, whereas proteins not identified in DRM were widely distributed in the cell membrane. Moreover, the results of immunodetection assays of the selected proteins were in agreement with the proteomic and microscopy analyses. While localization of proteins within foci does not necessarily involve active recruitment to lipid rafts and could implicate other elements such as the bacterial cytoskeleton, the combination of the identification of a given protein, by proteomic analysis, immunodetection, and fluorescence microscopy localization, strongly suggests that such protein could be a *bona fide* lipid raft resident. Nevertheless, the mechanism by which proteins are partitioned into membrane microdomains, as well as the functional consequences of this recruitment remains unclear and in need of further investigation.

Finally, an attracting issue in the study of bacterial lipid rafts is their lipid composition. In eukaryotic membranes, cholesterol increases membrane thickness and reduces its fluidity by improving the close packing of the longer and saturated acyl chains of sphingolipids. This promotes the segregation of sphingolipids from glycerophospholipids and leads to raft formation. However, most bacteria lack sterols and sphingolipids, and other specialized lipid species, such as farnesol [12], polyisoprenoid lipids (carotenoids) [9], or cyclic polyisoprenoid lipids (hopanoids) [51] have been proposed to functionally and structurally replace cholesterol in ordered membrane microdomains. Nevertheless, neither of these lipid species has been found to be present in *E*. *coli* membranes. Thus, a comparative lipidomic analysis between DRM and DSM fractions obtained from *E*. *coli* inner membrane is of foremost interest.

## Supporting information

S1 Fig Schematic work-flow for plasmids construction. (TIFF)

S1 Table DNA oligonucleotides used in this work. (XLSX)

S2 Table Total spectra counts obtained by LC/MS/MS and protein assignment. (XLSX)

S3 Table Relative abundance of inner membrane proteins in IM and DRM samples. (XLSX)

S4 Table Additional DRM enriched proteins and putative lipid raft-resident. (XLSX)
