# Supplementary figures and images for: Proteomic analysis of Escherichia coli detergent-resistant membranes (DRM)
Source: PLoS One. 2019 Oct 11;14(10):e0223794. doi: 10.1371/journal.pone.0223794 (PMC6788730; doi:10.1371/journal.pone.0223794)

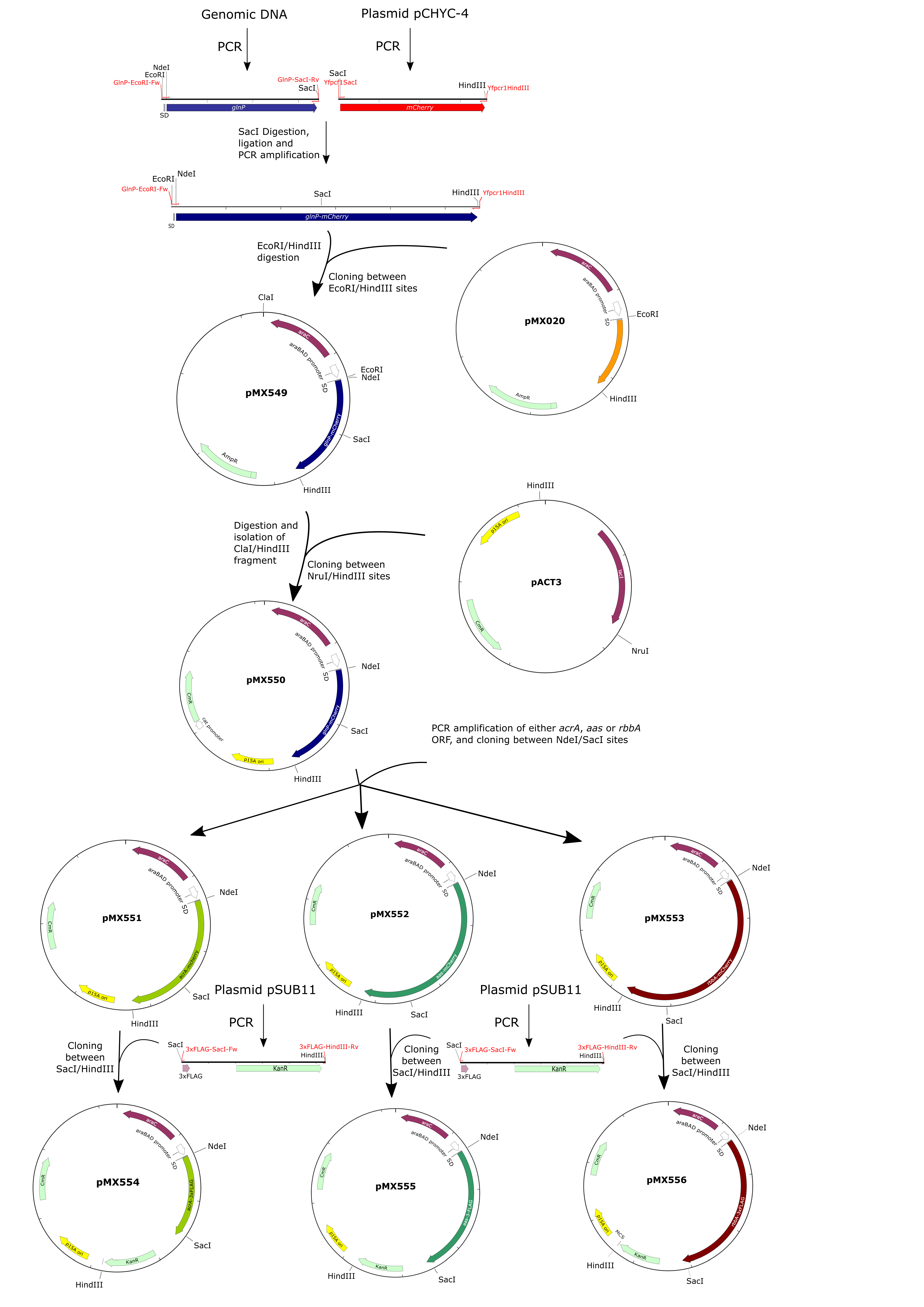

Supplement: S1 Fig — (TIFF) [file pone.0223794.s001.tiff]
